# Sugar and blood: the nutritional priorities of the dengue vector, *Aedes aegypti*

**DOI:** 10.1186/s13071-023-06093-5

**Published:** 2024-01-21

**Authors:** Frank Chelestino Tenywa, Jeremiah John Musa, Revocatus Musyangi Musiba, Johnson Kyeba Swai, Ahmad Bakar Mpelepele, Fredros Oketch Okumu, Marta Ferreira Maia

**Affiliations:** 1 Environmental Health and Ecological Sciences Thematic Group, Ifakara Health Institute, P.O. Box 74, Bagamoyo, Tanzania; 2 The Nelson Mandela African Institution of Science and Technology: School of Life Science and Bio-Engineering, P.O. Box 447, Arusha, Tanzania; 3 School of Medical Sciences, Faculty of Biology, Medicine and Health, The University of Manchester, 1.485 Stopford Building, Manchester, M13 9PL UK; 4 Faculty of Health Science, School of Public Health, University of the Witwatersrand, Johannesburg, South Africa; 5 KEMRI Wellcome Trust Research Programme, Centre for Geographic Medicine Research -Coast, PO Box, Kilifi, 230-80108 Kenya; 6 Centre for Global Health and Tropical Medicine, Nuffield Department of Medicine, University of Oxford, Old Road Campus Roosevelt Drive, Oxford, OX3 7FZ UK

**Keywords:** Attractive targeted sugar baits (ATSB), *Aedes aegypti*, Sugar-feeding, Blood-feeding, Dengue vectors

## Abstract

**Background:**

Sugar-feeding behaviour is essential for mosquito survival and reproduction, and has been exploited to develop new control strategies, such as the attractive targeted sugar baits (ATSB). This study examined the sugar-feeding habits of the dengue vector, *Aedes aegypti,* in semi-field conditions to determine the optimal timing (age) of sugar meals and whether the availability of sugar sources could affect blood-feeding by these mosquitoes.

**Methods:**

A series of paired-choice assays were conducted in which mosquitoes were allowed to choose between a sugar meal or a blood meal directly from a rabbit. Female 1-day-old mosquitoes were given meal choices in cages I–V and observed for feeding choice in only one cage every day for 5 days starting with cages I to V. The preference of *Ae. aegypti* to feed on sugar or blood and the effect of sugar source availability on blood-feeding was assessed at different chronological and physiological ages.

**Results:**

In the first 5 days post-emergence, there was no significant difference in mosquito preference for sugar or blood meals. However, after the first gonotrophic cycle, they had a greater preference for blood over sugar (odds ratio, OR [95% confidence interval, CI] = 9.4 [6.7–13.0]; *P* < 0.001). Nulliparous *Ae. aegypti* females (≤ 5-day-old mosquitoes) were less likely to blood-feed if both sugar and blood sources were concurrently available (OR = 0.06 [0.02–0.16]; *P* < 0.001).

**Conclusions:**

Newly emerged females of *Ae. aegypti* mosquitoes were equally likely to choose a sugar meal or a blood meal. However, after the first gonotrophic cycle, they had a greater preference for blood over sugar. Additionally, nulliparous female mosquitoes were less likely to blood-feed when both sugar and blood sources were available. These findings provide insights into the sugar-feeding behaviour of *Ae. aegypti* and can inform the development and optimization of new control strategies such as using ATSB.

**Graphical Abstract:**

**Figure:**
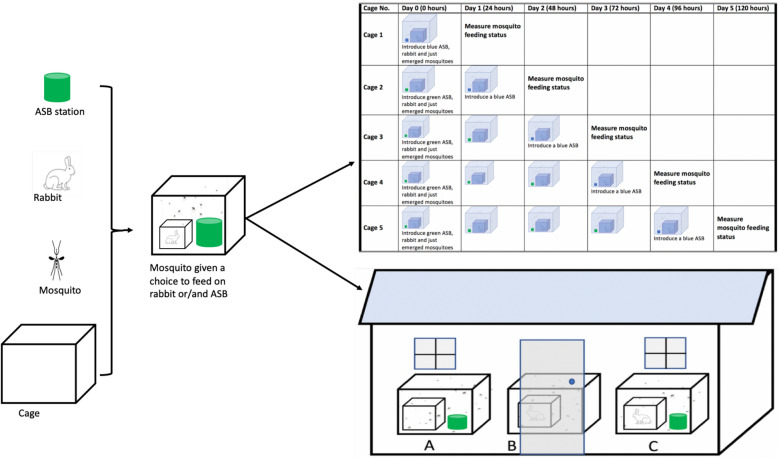

## Background

Both male and female mosquitoes feed on sugar from plants and fruits to enhance their survival, fitness, and reproduction [1]. While male mosquitoes feed exclusively on sugar, females feed on both sugar and blood [2–4], questing for sugar for energy supply and blood for egg development [5]. The blood meal also increases female mosquito survival [5, 6]. In some mosquito species, the sugar intake varies between nulliparous and parous stages [7, 8], highlighting the fact that the energy requirement is life-cycle-dependent.

While sugar-feeding is broadly essential for mosquito survival [1], females of *Aedes aegypti,* which transmit dengue fever and several other arboviruses, have been reported as capable of feeding exclusively on blood, which they use for energy supply as well as egg nourishment [6]. The mosquitoes may frequently imbibe multiple blood meals [9] to enhance their fitness and reproductive advantages [10, 11]. Blood-feeding frequency has also been shown to increase the pathogen transmission potential of some mosquitoes, particularly *Anopheles* species [12, 13], and the biting rate is a fundamental basis of vectorial capacity. However, the reported sugar-feeding exclusivity is not a rule of thumb, because some observations have shown that *Ae. aegypti* frequently feed on sugar [14–16], indicating that the exclusivity is not the choice of *Ae. aegypti* but rather an environmental-dependent fact. Therefore, whether *Ae. aegypti* commonly feed on natural sugar remains an inconclusive concept that perhaps needs further evidence.

Studies on the ecology of *Ae. aegypti* have shown that blood-feeding exclusivity may be a function of resource availability, and that in sugar-rich environments, the mosquitoes will feed on sugar even in the presence of blood sources [17, 18]. This has clearly been evidenced by some scholars who investigated the effect of attractive targeted sugar baits (ATSB) on *Ae. aegypti* depopulation and found that ATSB applied on vegetation decimated the *Ae. aegypti* population at a substantial level [19]. The drastic reduction of *Ae. aegypti* concurs with the findings from other studies [20–22] against other mosquito species. Also, Sissoko et al. [23] demonstrated that *Ae. aegypti* are responsive to natural sugar sources, and the ability to respond to the natural sugar sources depends on the plant’s odour as well as the volatiles produced [24]. On the other hand, Klowden [25] reported poor egg development when females were deprived of sugar, indicating that the carbohydrate source may actually be a dietary requirement of the female mosquito.

This study therefore examined the sugar-feeding habits of the dengue vector, *Ae. aegypti,* in semi-field conditions to determine the optimal timing of the sugar meals and whether the availability of sugar sources could affect blood-feeding by these mosquitoes.

## Methods

### Mosquitoes

Mosquitoes were obtained from a laboratory colony originally raised from wild-caught larvae from Bagamoyo, coastal Tanzania. Molecular analysis on the second filial generation of this colony using real-time polymerase chain reaction (RT-PCR) [26] confirmed the identity as *Ae. aegypti.* All experiments were conducted at Ifakara Health Institute’s facilities in Bagamoyo, using disease-free insectary-reared *Ae. aegypti*. The mosquitoes were reared at 27 ± 2 °C and 75 ± 20% humidity. Larvae were fed TetraMin^®^ fish food, while adults were maintained with 10% w/v sugar solution with 12:12 day/night light cycles. For egg laying, female *Ae. aegypti* aged 3–6 days were fed on bovine blood via a membrane.

### Experiment 1: assessing the preferences of newly emerged *Ae. aegypti* for sugar and blood meals

The feeding behaviour of female *Ae. aegypti* mosquitoes was observed for 5 days after their emergence. The experiments were conducted in an experimental hut inside a semi-field facility. Five metal cages (120 cm × 120 cm × 120 cm) with removable netting panels and a plywood base were positioned inside the hut. Each cage had a long sleeve on one panel which allowed the research team to release and collect mosquitoes using an aspirator [27]. A rabbit with a shaved back was kept in a smaller wooden cage (60 cm × 60 cm × 60 cm) placed within the larger metal cage. The rabbit's cage had wire mesh panels that allowed the mosquitoes to easily fly in and out, ensuring constant access to both sugar and blood.

**Fig. 1:**
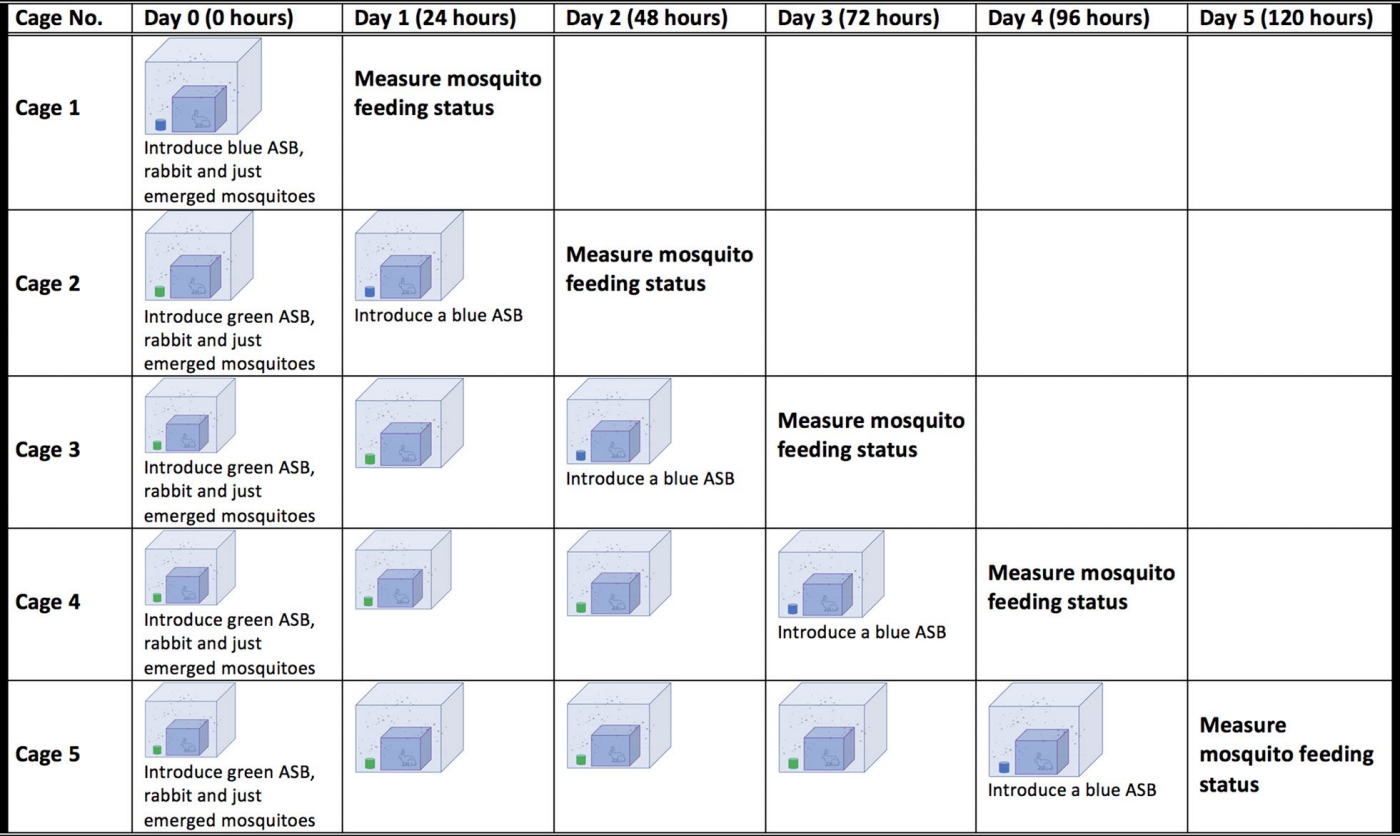
Feeding choices of *Aedes aegypti* between sugar and host blood meal. Sugar baits and rabbits were placed in large cages for female mosquitoes to choose their preferred meal, with results determined 24 h after introducing a blue-coloured sugar bait

Five sugar baits (SBs) were prepared without any toxicants, following the method described by Tenywa et al. [28]. A stock solution of sugar (10% w/v) was made and divided into two portions: one was dyed blue with a non-toxic food colouring dye (0.5% v/v). and the other was dyed green with a non-toxic food colouring dye (0.5% v/v). Each cage was assigned an experimental time period: cage I—24 h, cage II—48 h, cage III—72 h, cage IV—96 h, and cage V—120 h (Fig. 1). Forty newly emerged (0-day-old) female *Ae. aegypti* mosquitoes (naïve to both blood and sugar) were introduced into each of the five cages and kept there throughout their respective experimental time period.

A green-dyed SB was placed inside each cage until 24 h prior to each cage’s experimental time period, and then replaced with a blue-dyed SB (Fig. 1). The following day (24 h post-introduction of blue-dyed SB for cages II–V), all mosquitoes were collected using a Prokopack aspirator [29], killed in a freezer for 30 min, and observed for feeding status. This was done by squeezing their abdomens onto a white filter paper and checking for the presence of either food dye or blood: blue dye indicated feeding on sugar within the last 24 h; green dye indicated feeding on sugar within 24 h before the previous 24 h.

At the end of the respective experimental time periods, the rabbit was returned to the animal nursing house. The cage was thoroughly cleaned and left for the next experimental replicate. The rabbits were rotated between the cages to avoid bias due to any differential mosquito attractiveness, and eight experimental replicates were performed.

### Experiment 2: assessing the meal choices of *Ae. aegypti* after the first gonotrophic cycle

**Fig. 2:**
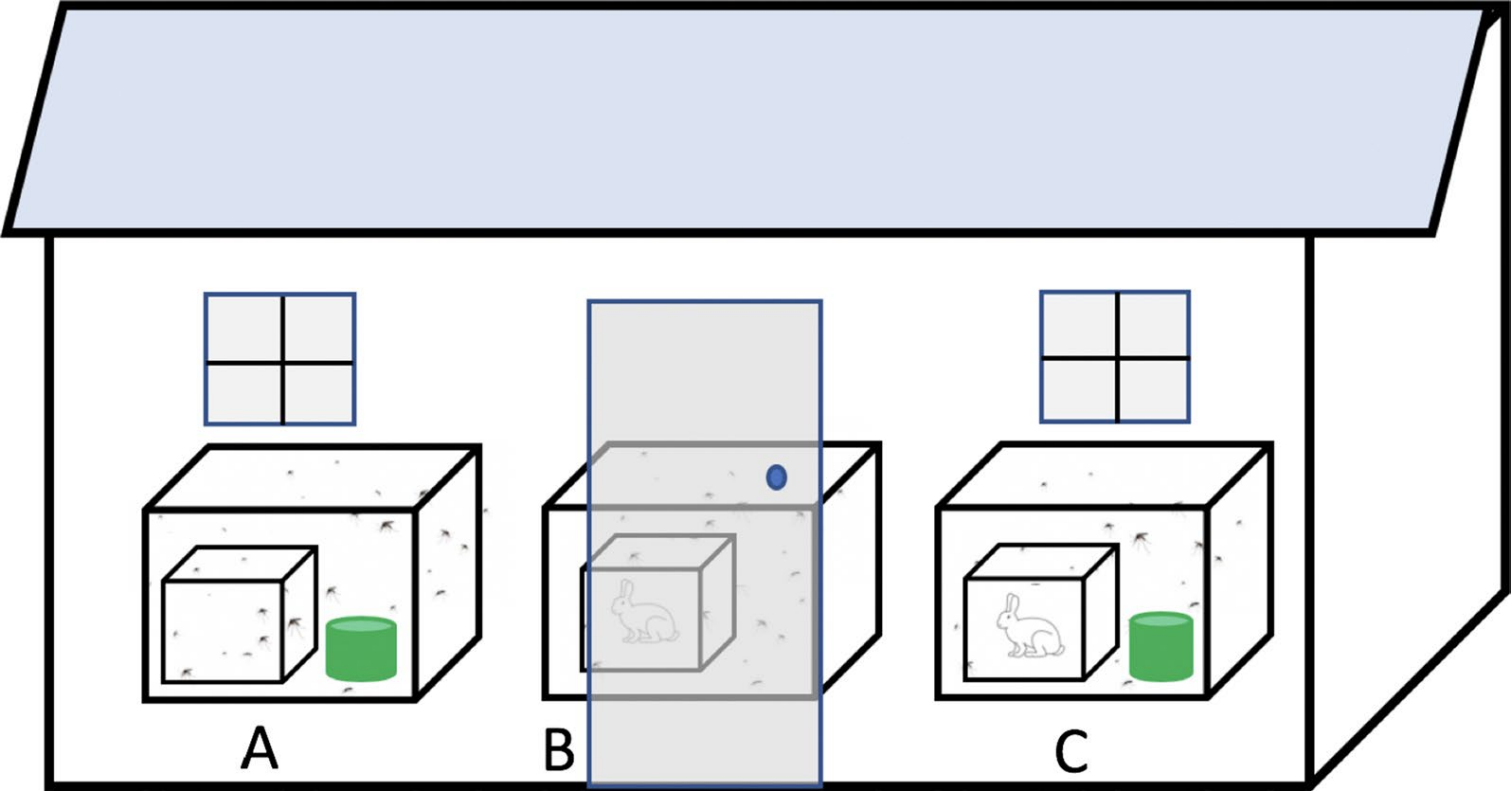
Experimental setup to study *Aedes aegypti* feeding behaviour with sugar and blood meals illustrated schematically. Cages were provided with sugar bait alone (**A**), rabbit alone as blood source (**B**), or a combination of both (**C**) for 24 h

Three-day-old female mosquitoes, given the opportunity to mate prior to use, were blood-fed on cattle blood through a membrane feeding system, and then 150 fully blood-fed mosquitoes were transferred to holding cages and kept in insectary conditions for 48 h to allow for digestion of the blood. Batches of 50 mosquitoes each were transferred to large cages containing a rabbit and a green-dyed SB (Fig. 2). Oviposition bowls were placed in each cage, and the presence of eggs was observed 24 h later. Upon observing the presence of eggs, the green-dyed SBs were replaced with blue-dyed SBs and left for 24 h. After 24 h, mosquitoes from each cage were collected using a Prokopack aspirator [29] and observed for feeding status by squeezing their abdomens onto a white tissue paper. The number of sugar-fed and blood-fed mosquitoes were determined. Ten replicates were performed.

### Experiment 3: assessing the effect of sugar availability on the blood-feeding behaviours of *Ae. aegypti*

Three large cages as described in previous experiments were used to determine whether limited sugar availability would affect the blood-feeding of nulliparous *Ae. aegypti*. In each set of cages, 40 blood-naïve *Ae. aegypti* females, aged 3–7 days and starved for 6–8 h, were released into each of the three cages. The mosquitoes were obtained from a mosquito-rearing cage where male mosquitoes were present to allow mating. The first cage contained only an SB, the second cage contained only a rabbit, and the third cage contained both a rabbit and an SB. The mosquitoes were allowed to choose the sugar meal, the blood meal, or both; 24 h post-release, they were collected using a Prokopack aspirator [29] and observed for feeding status by squeezing the abdomens as described earlier. Ten replicates were performed, with the positions of the cages being changed after each replicate to minimize potential sources of bias.

### Data analysis

All data obtained were analysed using Stata version 13 software (StataCorp LLC, College Station, TX, USA). For the first experiment, a generalized linear regression model with logit link was performed to determine the difference in the proportion of sugar-fed or blood-fed mosquitoes within the first 5 days post-emergence. The proportion of fed mosquitoes (sugar-fed, blood-fed and those which fed on both meals) were considered as dependent variables, while meal type was regarded as independent variable. Replicates (experimental days) and cage position were treated as covariates. Odds ratios (OR) and 95% confidence interval (95% CI) were obtained from the model. For the second and third experiments, a descriptive analysis and logistic regression were performed to compare the percentage mean of sugar-fed, blood-fed and mixed-fed mosquitoes at 95% CI before and after the first gonotrophic cycle. The odds ratios at a 95% confidence interval were estimated from the models.

## Results

### Preferences of newly emerged *Ae. aegypti* for sugar or blood meals

The proportions of *Ae. aegypti* mosquitoes which fed on blood only, sugar only or mixed meals were not statistically different over the first 5 days post-emergence (Table 1). However, on the fifth day post-emergence, mosquitoes were statistically more likely to take both sugar and blood than just blood (OR = 1.96 [1.17–3.26]; *P* = 0.010).

**Table 1:** Mean proportions and odds ratios for *Ae. aegypti* fed on sugar, blood or both

Days post-emergence	*N*	*n*	Mean proportion	Odds ratio	95% CI	*P*-value
Day 1						
Blood-fed	8	299	0.28	1	–	–
Sugar-fed	8	299	0.36	1.45	0.77–2.7	0.246
Mixed-fed	8	299	0.27	0.98	0.57–1.68	0.938
Unfed	8	299	0.08	0.26	0.14–0.46	< 0.001
Day 2						
Blood-fed	8	301	0.31	1	–	–
Sugar-fed	8	301	0.21	0.62	0.29–1.31	0.208
Mixed-fed	8	301	0.40	1.56	0.72–3.38	0.259
Unfed	8	301	0.08	0.22	0.09–0.54	0.001
Day 3						
Blood-fed	8	332	0.20	1	–	–
Sugar-fed	8	332	0.28	1.60	0.73–3.52	0.241
Mixed	8	332	0.33	2.08	0.91–4.72	0.079
Unfed	8	332	0.19	0.95	0.40–2.25	0.913
Day 4						
Blood-fed	8	304	0.23	1	–	–
Sugar-fed	8	304	0.27	1.22	0.69–2.14	0.497
Mixed	8	304	0.31	1.49	0.83–2.66	0.181
Unfed	8	304	0.19	0.83	0.34–2.00	0.674
Day 5						
Blood-fed	8	295	0.21	1	–	–
Sugar-fed	8	295	0.32	1.79	0.92–3.50	0.088
Mixed	8	295	0.34	1.96	1.17–3.26	0.010
Unfed	8	295	0.13	0.61	0.28–1.32	0.209

*N* = number of replicates, *n* = total number of released mosquitoes, 95% CI = 95% confidence interval

### Meal preferences of *Ae. aegypti* females before (experiment 3) and after (experiment 2) the first gonotrophic cycle

**Fig. 3:**
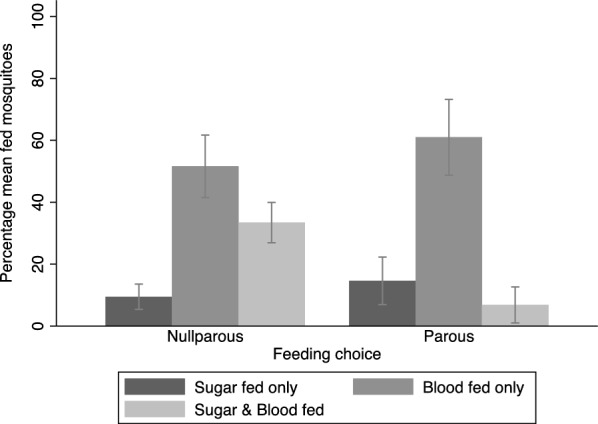
Proportion of *Aedes aegypti* meal preferences before and after the first gonotrophic cycle in a cage with both sugar and blood meals available

A significant number of *Ae. aegypti* preferred to feed on a blood meal over a sugar meal within 24 h before and after the first gonotrophic cycle (Fig. 3). Out of 385 mosquitoes given choices before the first gonotrophic cycle, 51.6% [40.2–63.0] fed on blood, 9.5% [4.8–14.1] fed on sugar, and 33.3% [33.4–46.1] fed on both blood and sugar. Moreover, after oviposition, 61% [46.9–75.0] fed on the blood meal, 14.6% [5.8–23.4] on sugar, and 6.8% [0.2–13.5] on both meal types (Fig. 3).

### Effects of sugar availability on the blood-feeding behaviours of *Ae. aegypti* before and after the first gonotrophic cycle

The presence of a sugar meal in close proximity appeared to influence the shift in blood-feeding preference for both nulliparous and parous *Ae. aegypti* females (Table 2). When offered both blood and sugar in the same cage, the number of nulliparous *Ae. aegypti* that fed on blood only was significantly lower (OR = 0.06 [0.02–0.16]; *P* < 0.001) than those in a cage that had been offered only the blood meal. This difference was however only marginal in parous mosquitoes (OR = 0.80 [0.57–1.03]; *P* = 0.08) (Table 2).

**Table 2:** Percentage blood-fed nulliparous and parous *Ae. aegypti* offered either blood meals only or both blood and sugar meals in the same cage

Parity status	Feeding preference	*N*	*n*	Percentage fed [95% CI]	OR	95% CI	*P*-value
**Nulliparous**	Blood-fed in a cage with only blood meal	10	389	99.0 [98.0–99.9]	1	–	
	Blood-fed in a cage with sugar and blood meal	10	385	85.0 [76.5–93.5]	0.06	0.02–0.16	< 0.001
**Parous**	Blood-fed in a cage with only blood meal	10	427	73.3[60.7–85.9]	1	–	
	Blood-fed in a cage with sugar and blood meal	10	443	67.8[54.8–80.8]	0.80	0.57–1.03	0.08

*N* = number of replicates, *n* = total number of released mosquitoes, 95% CI = 95% confidence intervals

## Discussion

The sugar-feeding habits of female *Ae. aegypti* mosquitoes vary depending on the stage of their life cycle. This study found that at the nulliparous stage, sugar and blood meals are both essential for *Ae. aegypti*. However, shortly after completing the gonotrophic cycle, blood meals become a higher priority over sugar meals. This is likely because the depleted protein reserves used during the gonotrophic cycle require additional protein to prepare for the next cycle. Mosquitoes require energy mainly from sugar sources for survival, fitness and fecundity [1, 3], which they require shortly after emergence. On the other hand, they need protein [2] for egg development. These functions indicate that the two meals are components which need to occur concurrently for the existence of the mosquitoes.

The sugar reserves from the sugar meal consumed before and during the gonotrophic cycle may explain why *Ae. aegypti* does not require additional energy from another sugar meal after the first gonotrophic cycle. This hypothesis aligns with findings that carbohydrates consumed by *Ae. aegypti* during the larval and early adult stages are usually stored and used during and after the gonotrophic cycle [30, 31]. The ability of *Ae. aegypti* to store carbohydrates may have enabled the mosquitoes to adapt to living in areas with low or inadequate sugar sources, and also influenced the anautogenous mosquitoes to predominantly feed on blood [32]. This adaptation to only blood meals increases their contact frequency with humans [33], which is in the transmission of vector-borne diseases. There is also evidence that mated *Ae. aegypti* tend to feed more on blood than the virgin, likely due to their readiness to lay eggs. However, studies have failed to identify a correlation between mating and mosquito blood-feeding rate [34]. Nevertheless, we did not dissect mosquitoes to observe mating plugs, and therefore we are unable to draw conclusions.

The study also showed that mixed feeding of sugar and blood meals is common in *Ae. aegypti* females, especially at the nulliparous stage. This highlights an important opportunity for developing interventions such as ATSB against *Ae. aegypti*, which has been effective against other species such as *Aedes albopictus* [20, 35, 36]. Since the mixed feeding observed in this study was in a controlled environment using insectary-reared mosquitoes, it remains to be determined whether the same behaviour occurs in wild populations when sugars are available.

The majority of nulliparous and parous *Ae. aegypti* that had been pre-fed on a sugar meal preferred to take blood over sugars when both options were constantly available. The observed findings may be because the mosquitoes already had enough sugar reserves and no longer needed additional energy, but instead needed protein sources for first and second gonotrophic cycles. Although the female *Ae. aegypti* that had previously fed on sugar preferred blood over sugar, mixed meals were favoured more at the nulliparous stage than the parous stage, indicating that younger mosquitoes require more energy than older mosquitoes.

When both sugar and blood sources were available, the mosquito blood-feeding rate was reduced considerably relative to when only a blood host was available. This reduction in blood-feeding due to the availability of a sugar source has important implications for vector control strategies using interventions like ATSB, as it suggests that it may also reduce blood-feeding frequency and thus have a significant impact on disease transmission and vectorial capacity [37]. A broad argument has been drawn that mosquitoes take sugar meals mainly for energy provision [38], but there is an additional advantage in that sugar meal intake reduces female mosquito blood-feeding [39], which impacts mosquito fecundity. Moreover, sugar intake is also reported to increase mosquito immune gene expression [40, 41], hindering pathogens from successfully invading the mosquito gut. However, these findings need further review to draw firm conclusions. Modelling studies have indicated that a minimum daily intake of sugar in the form of ATSB can significantly reduce malaria cases [42]; however, it is not yet known whether this is the case for arboviruses transmitted by mosquitoes*.* Although some researchers have reported that *Ae. aegypti* are exclusively blood-feeders [1], our findings refute the concept, concurring with other studies which investigated the same mosquito ecological behaviour and found that the mosquito species do feed on sugar [17, 18], implying that the tendency may be attributed to the availability of sugar sources.

While the key objectives of this study were achieved, one limitation was that the mosquitoes had to be provided a constant choice of blood and sugar meals, which was only possible with non-human blood sources. Thus, a rabbit was chosen as an alternative host.

## Conclusions

This study showed that the preference for sugar and blood meals in *Ae. aegypti* mosquitoes varies depending on the stage of the life cycle. While 1–5-day-old nulliparous females exhibited a similar affinity for both sugar and blood meals, older parous mosquitoes preferred blood meals over sugar meals. The early preference for sugar meals in mosquitoes may have important implications for the development of vector control strategies, such as ATSB, which could target mosquitoes that have not yet reached infective stages.

## Data Availability

The datasets generated during the study will be available on the Open Science Framework. https://osf.io/szy52/
